# DNA Repair—A Double-Edged Sword in the Genomic Stability of Cancer Cells—The Case of Chronic Myeloid Leukemia

**DOI:** 10.3390/ijms161126049

**Published:** 2015-11-18

**Authors:** Elzbieta Pawlowska, Janusz Blasiak

**Affiliations:** 1 Department of Orthodontics, Medical University of Lodz, 92-216 Lodz, Poland; elzbieta.pawlowska@umed.lodz.pl; 2 Department of Molecular Genetics, University of Lodz, 90-236 Lodz, Poland

**Keywords:** DNA repair, genomic instability, BCR-ABL1, chronic myeloid leukemia

## Abstract

Genomic instability is a common feature of cancer cells, which can result from aberrant DNA damage reaction (DDR). We and others showed that the well-known *BCR-ABL1* fusion oncogene, the cause of chronic myeloid leukemia, induced an increased production of reactive oxygen species (ROS) and conferred therapeutic drug resistance by suppression of apoptotic signaling, prolonged G2/M arrest and stimulation of several pathways of DNA repair. However, to protect from apoptosis, cancer cells may tolerate some DNA lesions, which may increase genomic instability. Moreover, BCR/ABL1-stimulated DNA repair might be faulty, especially non-homologous end joining in its alternative forms. Normal DNA repair can remove DNA damage and prevent mutations, reducing genome instability, but on the other hand, due to its imprecise nature, it may increase genomic instability by increasing the ratio of mutagenic DNA lesions. The example of BCR-ABL1-expressing cells shows that DNA repair can both increase and decrease genomic instability of cancer cells and understanding the mechanism of the regulation of these opposite effects would be helpful in anticancer strategies.

## 1. BCR-ABL1 and Chronic Myeloid Leukemia

**Figure 1:**
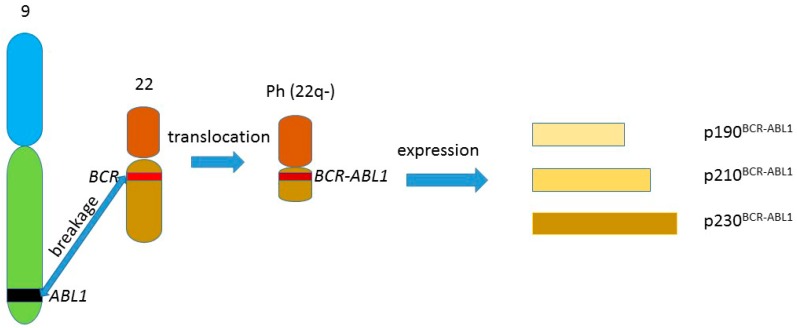
The t(9;22)(q34;q11) reciprocal chromosomal translocation producing the Philadelphia chromosome (Ph) (22q–), containing the *BCR-ABL1* gene, the expression of which can give three fusion proteins of different lengths due to alternative splicing of the BCR-ABL1 mRNA. The other product of the translocation—the 9q+ chromosome, is not included in the figure.

The t(9;22)(q34;q11) reciprocal chromosomal translocation produces the Philadelphia chromosome, containing juxtaposed fragments of the *BCR* and *ABL1* genes, forming the *BCR-ABL1* fusion gene. The product of this gene, the BCR-ABL1 protein, has a constitutive tyrosine kinase activity, which promotes cell proliferation in the absence of growth factors. The expression of the gene can give three forms of BCR-ABL1 due to alternative splicing (Figure 1). This distinct chromosomal abnormality was causatively linked with chronic myeloid leukemia (CML), which was the first human cancer associated with chromosomal aberration (reviewed in [1]). BCR-ABL1 plays also a role in the genomic instability of CML cells, which will be discussed later.

**Figure 2:**
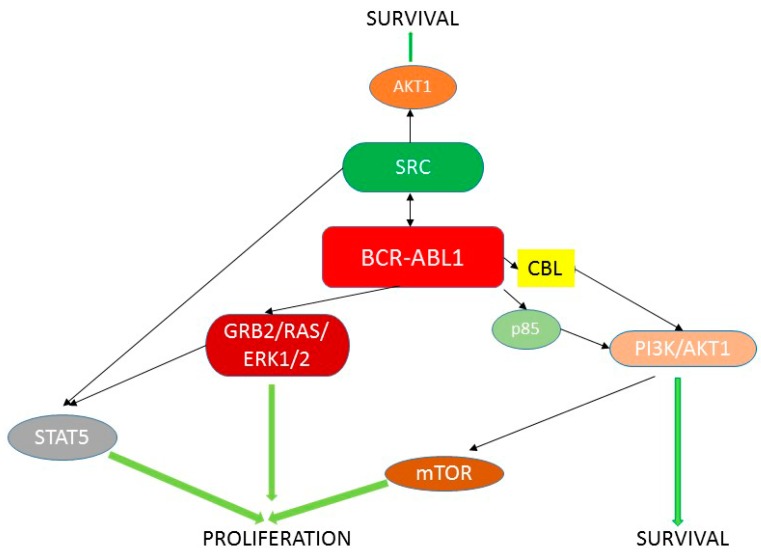
Some BCR-ABL1 signaling pathways important for cancer transformation (adapted from [2]).

It is believed that the cancer phenotype conferred by BCR-ABL1 is mainly due to its anti-apoptotic, pro-survival properties, but this protein is involved in so many signaling pathways that the complete mechanism of BCR-ABL1 carcinogenicity can be much more complex. Some of the signaling pathways important for the leukemogenic effect of BCR-ABL1 are presented in Figure 2.

Expression and activity of BCR-ABL1 is also important in other cancers, including gastrointestinal cancer and a subset of acute lymphoblastic leukemia [3,4]. CML is considered as a disease derived from hematopoietic stem cells and progressing in three distinct phases: chronic phase (CP), associated with the expansion of myeloid progenitor and seemingly normal differentiation, accelerated phase (AP) and blast crisis/phase (BP), which is usually fatal [5]. Although BCR-ABL1 is responsible for the cancer phenotype in CML cells, the mechanisms involved in the CML progression to BP, drug resistance and disease relapse are not completely clear.

Imatinib mesylate (imatinib, STI571, Gleevec), a tyrosine kinase inhibitor (TKI) is the first successfully applied drug of targeted cancer therapy and it revolutionized the treatment of CML. However, imatinib resistance became an emerging problem, which was only partly resolved by second and third generation TKIs, including nilotinib, dasatinib and bosutinib (reviewed in [6]). The mechanism of action of imatinib is based on its interaction with the nucleotide-binding site of the active site of BCR-ABL1, preventing ATP binding, which is a cofactor necessary for BCR-ABL1 activity [7].

TKI-resistance in CML can be primary or secondary (acquired). Several mechanisms can underlie primary resistance to imatinib, including the drug export by P-glycoprotein, binding of imatinib in plasma by α1-acid glycoprotein, amplification of the *BCR-ABL1* gene and its altered expression, underlined by genetic and epigenetic mechanisms, and inducing of BCR-ABL1-independent pro-survival signaling pathways (reviewed in [8]). Secondary resistance to imatinib is acquired during CML therapy and usually results from mutations in the *BCR-ABL1* gene, which render BCR-ABL1 resistant to treatment with TKIs [9]. Secondary imatinib resistance is considered as one of the first signals of progressing CML into AP and BP and is linked with a short survival period [10].

As mentioned, CML progression to advanced stages has not been completely explained yet, and several mechanisms involved in this effect are considered. Some research suggests that this process can be associated with increased genomic instability of CML cells during TKI therapy [11]. Genomic instability, as will be discussed later, is a common feature of cancer cells, but its extent can increase with the CML progression, and advanced CML is associated with increased number of mutations in the *BCR-ABL1* gene.

One of the candidates to play a role in CML progression is the transcription factor STAT5, for which marked activation was observed in CML and can be considered as another hallmark of this disease [12]. However, this kind of STAT5 activation can be attributed to a direct action of BCR-ABL1 [13,14]. A high level of STAT5 was observed in CML patients in disease progression to advanced stage and a positive correlation between STAT5 expression and the occurrence of mutations in the *BCR-ABL1* gene was observed [15]. Therefore, STAT5 can be closely related to CML progression, sensitivity of CML cells to TKIs and ROS production. Several other signaling pathways, including Akt, PI3K and Wnt/β-catenin, can be involved in CML progression [16,17,18].

## 2. DNA Repair in Cellular DNA Damage Response

**Figure 3:**
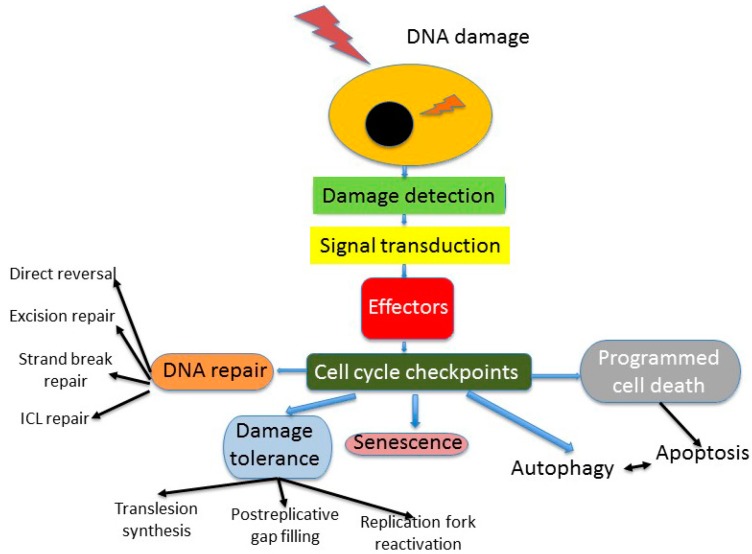
Some aspects of DNA damage response (DDR). DNA damage can be induced exogenously or originate from an endogenous insult. Cell cycle control is exerted only in proliferating cells, since terminally differentiated, quiescent and senescent cells, as well as some other kinds of cells, do not present this control. Stem cells can display some other kinds of DDR, programmed cell death is represented only by apoptosis, for which association with autophagy is not fully known. ICL—interstrand crosslink.

DNA repair is an essential component of the cellular DNA damage response (DDR), a reaction of the cell to deal with endogenous and exogenous genomic insults, which can interfere with the process of transfer of genetic information, disturb the cell cycle, and induce cancer transformation and cell death (Figure 3). Besides DNA repair, DDR includes also mechanisms of tolerance, various modes of programmed cell death and autophagy, which all are associated with cell cycle control [19]. Although DNA repair may be the most important in DDR and may lead to complete recovery of the cell, it may have various efficacy and accuracy.

In general, DNA repair is considered as error-free (or faithful), and error-prone (or faulty). Faulty DNA repair, sometimes referred to as misrepair, is not a repair *per se* and can lead to more detrimental consequences, than those resulting from leaving damaged DNA unrepaired [20]. Misrepair may be associated with recovering of DNA integrity, but DNA processed in this manner shows changes in the sequence, including lack of an original fragment, compared with native DNA. In humans, this can be observed in non-homologous end joining (NHEJ) and its variants, in which DNA double strand breaks are repaired by processing DNA ends, making them a substrate to DNA ligase [21]. This process can lead to the loss of DNA sequences. On the other hand, impreciseness of NHEJ in the opening of DNA hairpin structures contributes to the diversity of the immunological system [22].

DNA repair plays a special role in DDR in cancer since cancer cells, including cancer stem cells, are characterized by considerable levels of genomic instability [23]. Cancer transformation is a complex process with sequential genetic and epigenetic events and after the initiation each event can accelerate the next one. Genetic events are mainly DNA damages, which are not repaired or are misrepaired and turned into mutations supporting cancer transformation. However, these mutations may be casual for cancer development, but do not necessarily contribute directly to genomic instability of cancer cells. Instead, genomic instability, characterized by an increased general ratio of mutations, increases the possibility of cancer-specific mutations. Two main categories of genes which are associated with cancer transformation—oncogenes and tumor suppressors, can lead to cancer when mutated. And they are mutated when premutagenic DNA damage is not repaired. However, when there are mutations in DNA repair genes, the chance of such events increase, contributing to increased genomic instability.

## 3. Genomic Instability—A Common Feature of Cancer Cells

**Figure 4:**
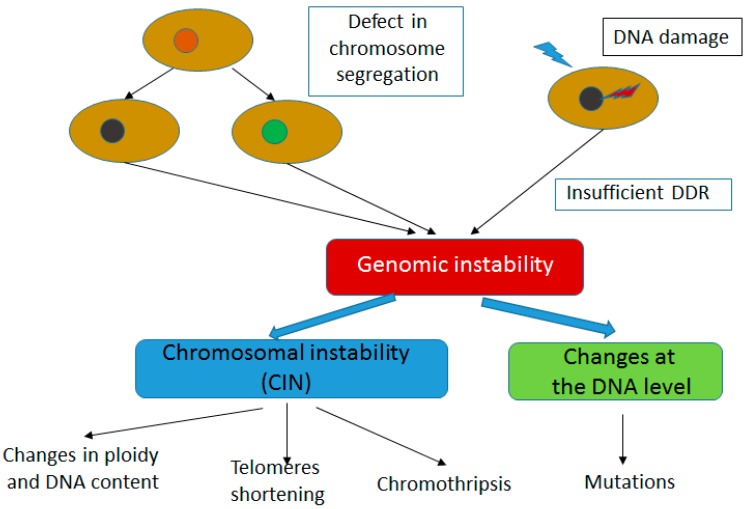
Genomic instability can result from defective chromosome separation, insufficient DNA damage response (DDR) or both. When the extent of endogenous or exogenous DNA damage exceeds the potential of DDR, some DNA damage can remain in the form of chromosomal aberration or changes in DNA sequence. Epigenetic mechanisms contributing to genomic instability are not presented.

Each human nucleated cell copies with more than 10^4^ damages to its genome from endogenous processes and various number of DNA lesions resulting from exogenous sources [24]. Genomic instability is understood as a state of the genome, in which an increased extent of DNA damage occurs (Figure 4). This can result from increased rate of DNA damage induction or aberrant DDR or both. DNA damage can be seen on the chromosome level, e.g., double strand breaks can lead to a chromosomal translocation, production of fusion chromosomes and this kind of genomic instability is associated with DNA disintegration. This likely leads to misunderstanding of the term genomic instability as genomic integrity. However, genomic instability can be also associated with changes in ploidy, *i.e.*, the number of normal chromosomes [25]. This kind of instability can be relatively easily detected in the cell with classical cytogenetic tools.

Mitosis requires several events to complete, which, if disturbed, can contribute to genomic instability. They include chromosome condensation, sister chromatid cohesion, kinetochore assembly and attachment, centrosome duplication and separation, spindle formation and chromatid segregation, but it is out of the scope of this review to deal with these issues in more details (reviewed in [26]). It seems that genomic instability manifested on the chromosome level can result from changes in the structural maintenance of chromosomes (Smc) proteins, regulating almost all aspects of chromosome metabolism (reviewed in [27]). In humans, there are at least six members of the Smc family of proteins, Smc1–6, forming three heterodimers and they may be important for genomic instability as Smc5 and Smc6 can form a complex playing an important role in DNA repair [28]. Moreover, amino acid composition and overall architecture of Rad50, a component of the MRN (Mre11-Rad50-Nbs1) complex, which is crucial for DNA double strand breaks (DSBs) repair signaling, make it a Smc-related protein [29]. The Smc2 and Smc4 members of the family are essential for chromosome condensation, as they form the condensing complexes, whereas Smc1 and Smc3 are critical for proper chromosome separation, as they are essential parts of the cohesin complex, involved in keeping sister chromatids at close proximity during mitosis [30,31].

Smc1 is involved in DSBs repair by homologous recombination (HRR) and its involvement in human NHEJ is possible [32,33,34]. Moreover, the Smc1/3 complex is recruited to DSB sites by the Smc5/6 dimer [33]. Additionally, cohesin can be involved in DNA damage checkpoint activation, especially in intra-S-phase checkpoint in response to UV, ionizing radiation and carcinogenic chemicals [35,36]. It was reported that overexpression of Smc4 was associated with colorectal cancer [37]. It is interesting that changes in ploidy may be associated with DNA damage, as we observed an increased extent of DNA damage in individuals with Down syndrome, compared to normal controls [38]. Down syndrome, a non-cancer disease associated with trisomy 21, is considered as a cancer-prone disease, at least for leukemias, as the cancer ratio in this disease is higher than average and this association can be underlined by the association between genomic instability on chromosome and DNA levels [39].

Chromothripsis is understood as a high number of rearrangements of DNA sequences resulting from chromosome shattering. Resulting fragments can then be aligned in a random fashion, resulting in a catastrophic event [40].

## 4. DNA Repair in Cancer Cells

DNA damage is essential for cancer transformation, especially in its initiation and progression stages. Therefore, such transformation can be associated with impairment in DNA repair or, in general, in DDR. Many cancers show defects in DNA repair proteins, especially those important for the repair of DSBs (reviewed in [41]). DSBs are of particular significance for cell fate, as they are produced physiologically as intermediates to form essential components of the immunological system during V(D)J recombination and class switch recombination and are indispensable in meiosis. However, aberration of some physiological processes, including reactive oxygen species (ROS) signaling, DNA replication and action of some enzymes, including the recombination-activating genes (RAGs) and DNA topoisomerase II, can lead to DSBs induction.

DSBs are causative for chronic myeloid leukemia, in which the cancer phenotype is associated with the *BCR-ABL1* oncogene, resulting from the reciprocal chromosomal translocation t(9;22)(q34;q11) initiated by DSBs.

The major pathways of DSBs repair are HRR and NHEJ, which may operate in several variants. The choice between HRR and NHEJ depends on the cell cycle phase and chromatin conformation, its regulation is not completely known and many proteins, including 53BP1, BRCA1 (breast cancer type 1 susceptibility protein) and WRN (Werner syndrome helicase), can be involved (reviewed in [42]). HRR is usually an accurate process, with the involvement of many proteins, including the MRN complex, RPA (replication protein A), RAD51 and its paralogs, BRCA1/2 and others, while the Ku70/80 dimer, DNA-PK_CS_ (catalytic subunit of DNA-depended protein kinase), Artemis, Ligase IV complexed with XRCC4 (X-ray repair cross-complementing protein 4), and Cernunnos (XLF) are essential for the canonical form of NHEJ (reviewed in [43,44]).

Deficiency in the activity of several HRR and NHEJ proteins were observed in many cancers, and they can be broadly divided into direct and indirect. The former include changes in the genes encoding these proteins, including mutations and amplification, whereas the latter are associated with the aberrant expression of those genes, resulting from changes in other genes/proteins. For instance, defects in RAD51, a protein crucial for HRR, were observed in pancreatic and breast cancer, some leukemias and sarcomas. On the other hand, malfunctioning of DNA-PKCS, an essential component of canonical NHEJ, was reported in gastric, breast, lung and esophageal cancers [40]. However, it is not clear, whether these defects are the reason or a consequence of cancer and in certain cases both scenarios should be taken into account.

The most common familial cancers, the hereditary non-polyposis colorectal cancer (HNPCC) and familial breast cancer, are associated with mutations in DNA repair proteins. HNPCC can be linked with mutations in several mismatch repair (MMR) genes, including *hMSH2*, *hMSH6*, *hMLH1*, *hMLH3*, *hPMS2*, whereas about 5% of all breast cancer is associated with *BRCA1/2* mutations [44,45]. Therefore, these DNA repair proteins are of a diagnostic and therapeutic value in those diseases. In general, DNA repair can be targeted in cancer, as ionizing radiation and many anticancer drugs damage DNA, thus decreasing DNA repair in cancer cells can result in increased efficacy of cancer therapy. Synthetic lethality with DNA repair genes opens new strategies in anticancer therapy [46]. However, radiation and drug resistance is frequently observed in cancer and it can be associated with mutations in DNA repair genes [47].

Given genomic instability is a common feature of cancer cells and it is associated with the increased ratio of mutations, it is somehow surprising, that cancer cells can develop resistance to DNA-damaging drugs. However, this apparent paradox is explained by the observations that drug resistance can also be associated with mutations. Therefore, normal DNA repair can remove DNA damage induced by anticancer drugs and affecting cancer evolution, but aberrant DNA repair can contribute to acquiring mutations, thereby underlying the drug resistance of cancer cells.

Several signaling pathways, overactivated in cancer cells, can affect DNA repair, including Hedgehog, PI3K, TLR, Akt, mTOR and others [48,49,50,51]. Again, it is not clear whether crosstalk between proteins of those pathways with DNA repair proteins is the reason or a result of cancer transformation. This concerns also BCR-ABL1 signaling in CML cells.

## 5. BCR-ABL1 Can Induce Genomic Instability Independently of Its Leukemogenic Effect

Results of experiments with BCR-ABL1-expressing transgenic mice showed that they had a higher spontaneous mutation rate than control animals, even before clinical onset of leukemia [52]. These observations, validated with imatinib, suggest that BCR-ABL1 can induce genomic instability independently of acquiring a cancer phenotype [53]. In the initial efforts to explain this hypothesis, two models were considered, as summarized by Illaria [14]. One assumed that BCR-ABL1 changed the cell ability to repair DNA damage. This model was supported by the involvement of normal *c*-ABL1 in DNA repair [54,55,56]. Therefore, mutated forms of the BCR-ABL1 kinase can differently influence DNA repair than its normal counterpart and affect genomic stability. However, *c*-ABL1 may not be indispensable for DNA repair as cells deprived of this protein retained the ability to remove DNA damage [57,58]. Moreover, those cells displayed normal mechanisms of cell cycle control. Therefore, the possible involvement of BCR-ABL1 in DNA repair might not be limited to interference with the function of normal *c*-ABL1, but also include more active effects, as involvement in the interaction between cell cycle regulators and apoptotic pathways [59,60].

The other model of inducing genomic instability by BCR-ABL1 does not assume its direct interference with DNA repair processes, but proposes that this kinase can directly induce mutations at the extent exceeding the repairing capacity of the cell [61]. This increase of mutation frequency can be associated with the interaction of BCR-ABL1 with DNA polymerase β, an error-prone form of DNA polymerase, as elevated expression of this enzyme was observed in BCR-ABL1-positive cells [62].

Several studies performed in the Skorski lab suggest that both increased mutagenicity of BCR-ABL1 *per se* and its interfering with DNA repair may contribute to enhanced genomic instability of BCR-ABL1-positive cells [63,64,65,66].

## 6. Role of BCR-ABL1 in Mutagenesis—ROS Production and Unfaithful DNA Repair

Sattler *et al.* showed that BCR-ABL1-expressing cells displayed a higher level of ROS [67]. The causative association between BCR-ABL1 activity and ROS production was evidenced by using imatinib. On the other hand, pyrrolidine dithiocarbonate and *N*-acetylcysteine, ROS-scavengers, decreased BCR-ABL1 activity, resulting in a decreased phosphorylation of its substrates. However, hydrogen peroxide stimulates the kinase activity of BCR-ABL1. These results led to the conclusion that increased ROS production was attributed to an enhanced BCR-ABL1 signaling, likely by the inhibition of cellular protein phosphatases.

Several mechanisms are considered to contribute to the modulation of ROS levels by BCR-ABL1. As mitochondria are a potent producer of cellular ROS, they are a natural candidate to be involved in this effect. It was shown that inhibition of the mitochondrial respiratory chain decreased ROS levels in BCR-ABL1-expressing cells [68]. ROS production by BCR-ABL1 can be stimulated by Rec2 GTPase, an enzyme regulating mitochondrial membrane potential and electron transfer in the mitochondrial respiratory chain complex III (MRC-III) in CML cells [65]. That study suggests that the Rac2-MRC-II-BCR-ABL1 is involved in genomic instability of both leukemia stem and progenitor cells (LSCs and LPCs). This is important as it leads to the conclusion that ROS-induced genomic instability in CML cells begins in LSCs and progresses in LPCs, which was supported by the observation that mutations in the *BCR-ABL1* gene associated with TKI resistance were observed in CP CML in the CD34+ CD38− (stem) and CD34+ CD38+ (progenitor) cells [69].

BCR-ABL1 activates Akt signaling that inhibits the forkhead O transcription factor (FOXO), which is crucial for resistance to oxidative stress in physiological conditions [70]. It was shown that FOXO played an essential role in the maintenance of LCSs with the involvement of tumor growth factor-β (TGF-β) [71].

If ROS are not scavenged, they can damage DNA and their continuous production, associated with a steady activation of BCR-ABL1, may lead to the accumulation of DNA damages, which are likely to turn into mutations. These mutations directly contribute to increased genomic instability in CML cells. Moreover, the accumulation of mutations increases the chance of their occurrence in DNA maintenance genes, including DNA repair genes, further enhancing genomic instability. Therefore, BCR-ABL1-expressing cells, including CML cells, are likely to have enhanced levels of ROS, which can induce DNA damage, which, if not repaired, may contribute to genomic instability in these cells. Therefore, functioning of DNA repair in BCR-ABL1 cells may be crucial for their genomic stability and, therefore, CML progression. Our initial reports suggested that BCR-ABL1 could stimulate certain pathways of DNA repair, which might contribute to the resistance of these cells to anticancer drugs and radiation [71,72,73,74,75,76,77]. As mentioned, BCR-ABL1 could directly induce an increase in the frequency of point mutations and this effect was reversed by imatinib [54]. However, dysregulation of DNA repair in CML cells would result in the altered ability of these cells to repair even spontaneous mutations, without an extra ROS production, mediated by BCR-ABL1, and in this way contributing to genomic instability. As *c*-ABL1 kinase can play a role in effective DNA repair [77,78], it could be assumed that its mutated form in the BCR/ABL1 protein can disturb its normal role in DNA repair, which can contribute to genomic stability. However, *c*-ABL1 is also involved in cell cycle regulation to repair DNA, interacting with p53 and RAD51 [79,80]. Therefore, the altered function of *c*-ABL1 in DNA repair in the BCR-ABL1 kinase may not be related to its involvement in the execution of DNA repair, but rather with its regulation by the cell cycle control. This is supported by the observation that *c*-ABL1 is not indispensable for DNA repair.

As mentioned above, CML progression to the advanced phase is associated with increased expression of STAT5, which can stimulate ROS production by regulating transcription of gene(s), which products are involved in this effect [15]. However, this was observed only in *BCR-ABL1*-expressing cells, suggesting the presence of a feed-forward loop, in which BCR-ABL1 increases ROS production and self-mutagenesis, contributing to CML progression in a STA5-dependent manner.

## 7. Aberrant DNA Repair in BCR-ABL1-Expressing Cells

Expression of BCR/ABL1 induces ROS, which can damage DNA, leading to alterations in the DNA structure, including DSBs [66,73,74,81,82]. It was shown that CML cells responded to increased DNA damage with increased DNA repair [81,83]. However this enhanced DNA repair can be unfaithful and lead to increased genomic instability. This can be particularly important in the repair of DSBs, as they are among the most serious DNA damages and if not repaired or misrepaired, can lead to chromosomal aberrations, cancer or death. NHEJ is a major DNA repair pathway dealing with DSBs and is dominant in higher eukaryotes, at least in G1 and early G2 phases [84].

Although NHEJ can operate without use of any homology, it can utilize short regions of microhomology, usually ranging 1–4 bp. However, the use of microhomology results in deletions, usually not exceeding 20 bp, which are consequences of the error-proneness of this DNA repair system. Therefore, the more DNA damage, the higher the chance for its increase and in this way, enhancing genomic instability. This can be especially important for BCR-ABL1-expressing cells, as it was shown that myeloid leukemia cells demonstrated constitutive DNA damage, which is targeted by key NHEJ components, the Ku70 and Ku86 proteins [81]. Therefore, enhanced rate of DNA repair, which is not positively correlated with its efficacy, can contribute to genomic instability of cancer cells. On the other hand, this DNA repair can suppress or even prevent cancer transformation by repairing DNA damage in genes important for acquiring malignant phenotype. Therefore, it is possible that both NHEJ deficiency and its overactivity can result in increased genomic instability in cancer cells. Therefore, if BCR-ABL1-expressing cells show an increased extent of constitutive DNA damage, they can increase their genomic instability by the recruiting NHEJ components to the sites of damage. Additionally, the increase in the instability may be accelerated through a direct stimulation of the NHEJ pathway by BCR-ABL1. In the first study showing overactive NHEJ in human CML cells, an increased amount of errors, manifested by deletions in a test plasmid, was observed [83]. In that study, Ku70 and Ku86 were identified as responsible for the unfaithful NHEJ. As no difference in the expression of key NHEJ elements, including Ku70/86, was detected, the observed effects might results from the changes of structural and functional properties of Ku proteins [84].

A variant of Ku86 protein was observed in human promyelocytic leukemia HL-60 cells, displaying different DNA-binding activity than its wild-type counterpart [85]. This variant can change both activity and fidelity of NHEJ. Therefore, BCR-ABL1 can be involved in the forming of the Ku with changed properties and the mechanism underlying this involvement is not clear. However, several hypotheses can be considered due to the participation of BCR-ABL1 in many signaling pathways [86]. The kinase may interfere with the transcription of the *Ku* gene, leading to its mutation(s) resulting in altered activity of the Ku protein(s). Also translation of the Ku transcript and posttranslational modification of the Ku70/86 proteins can be affected by BCR-ABL1, but these speculations need experimental research.

The results obtained by Gaymes *et al*., indicate that most deletions observed in CML cells resulted from joining of distant regions of microhomology by NHEJ [83]. Therefore, this suggests that in BCR-ABL1 cells, microhomology-dependent NHEJ is preferred. However, it should be taken into account that this mode of NHEJ is usually associated with Ku absence [87].

The expression of several other NHEJ proteins was reported to be altered in CML. Those include Artemis and DNA ligase IV, essential in canonical NHEJ and downregulated in CML, as well as WRN and DNA ligase IIIα, which function in an alternative NHEJ pathway, and were upregulated in CML [88]. Interestingly, the involvement of DNA-PK_CS_ (another essential protein in canonical NHEJ), in NHEJ-dependent genomic instability in BCR-ABL1+ cells seems to be contradictory, which can result from different CML models [89,90,91].

Recently it was shown that c-MYC, a target for the BCR-ABL tyrosine kinase signaling activity, played a role in the transcription activation and following expression of the DNA ligase IIIα (*LIG3*) and poly ADP ribose polymerase 1 (PARP1) in leukemic cells activated by tyrosine kinases [92]. These proteins play an important role in aberrant alternative NHEJ observed in these cells and CML patients. The main conclusion from these studies was that BCR-ABL1 induced c-Myc expression, which led to genomic instability through increased expression of proteins essential for error-prone alternative NHEJ.

The results obtained for NHEJ functioning in BCR-ABL1-positive cells should be also viewed in a broader context as it was reported that Ku86 suppressed chromosomal aberration and cancer transformation in mice as Ku86 double mutants showed chromosomal instability and significantly higher frequency of B-cell lymphoma [93]. Therefore, disturbances in Ku activity, observed in BCR-ABL1-expressing cells may be just one aspect of the role of this oncoprotein in genomic instability, exerted by its involvement in regulating DNA repair or cellular reaction to DNA damage.

## 8. Conclusions and Perspectives

**Figure 5:**
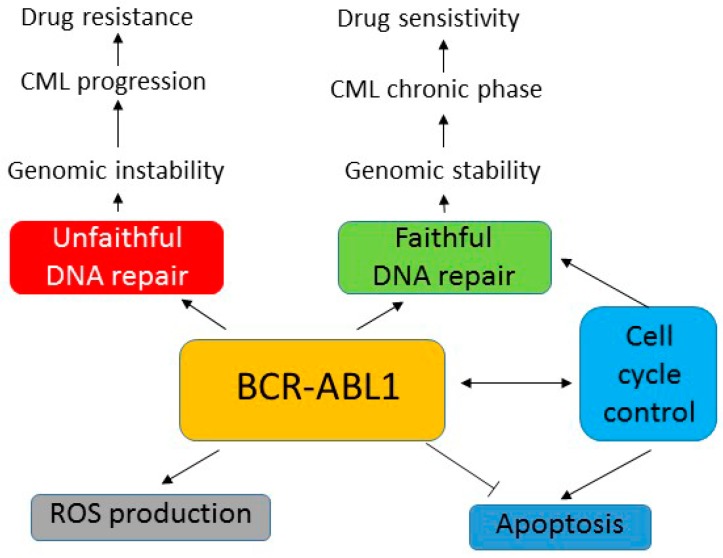
Some aspects of the involvement of BCR-ABL1 in the DNA damage response. Anti-apoptotioc effect of BCR-ABL1 is fundamental for conferring chronic myeloid leukemia (CML) phenotype, but the mechanism of progression of CML to blast phase, usually associated with therapeutic drug resistance, is largely unknown. Some experimental data suggest that BCR-ABL1 stimulates the production of reactive oxygen species and unfaithful DNA repair, which may contribute to genomic instability, disease progression and drug resistance.

Chronic myeloid leukemia cells produce an excess of reactive oxygen species in a BCR-ABL1-dependent manner, which stimulates mutagenesis in these cells. This process of self-mutagenesis is reinforced by increased faulty DNA repair, again dependent on BCR-ABL1. This leads to increased genomic instability of CML cells, which is associated with cancer phenotype of these cells, but can be independent of the leukemogenic effect of BCR-ABL1. Increased genomic instability can lead to accumulation of mutations in the *BCR-ABL1* gene, which can result in resistance to imatinib and other tyrosine kinase inhibitors (Figure 5). This genomic instability would be reduced by effective DNA repair and other mechanisms of DDR, which could result in reversing TKIs resistance and milder cancer phenotype of CML, hampering their progression into BP.

Therefore, in the case of TKI-resistant patients, not only anti-apoptotic properties of BCR-ABL1, mainly responsible for its carcinogenic nature, but also BCR-ABL1 activities associated with its pro-mutagenic character can be targeted. In general, two main pathways can be considered in this respect. The first is associated with the extent of ROS, for which production is stimulated by BCR-ABL1, and can be carried out by the supply of low molecular weight antioxidants, including free radicals scavengers, and stimulation of the activity of antioxidant enzymes. Pathways associated with increased ROS production by BCR-ABL1, including that stimulated by STAT5, can be blocked and/or modified, to limit ROS production. Studies on both of these issues are in progress. A second direction of research is linked with error-prone DNA repair stimulated by BCR-ABL1. Further study on signaling pathways involved in this process, such as recently identified c-MYC, can be helpful in identification of potential therapeutic targets in TKI-resistant CML cases. Such a study should primarily focus on the NHEJ mechanism, as this DNA repair pathway is strongly associated with faulty DNA repair in CML cells. However, due to the multi-pathway involvement of BCR-ABL1 in cellular signaling, addressing this issue needs further basic research before their results may be translated for clinical applications.
